# Efficacy and Safety of Ultrasonic Bone Curette‐assisted Dome‐like Laminoplasty in the Treatment of Cervical Ossification of Longitudinal Ligament

**DOI:** 10.1111/os.12858

**Published:** 2021-01-05

**Authors:** Baifeng Sun, Chen Xu, Shenshen Wu, Yizhi Zhang, Huiqiao Wu, Min Qi, Xiaolong Shen, Wen Yuan, Yang Liu

**Affiliations:** ^1^ Department of Spine Surgery, Changzheng Hospital Naval Medical University Shanghai China

**Keywords:** Axis, Dome‐like laminectomy, Laminoplasty, OPLL

## Abstract

**Objective:**

To assess the efficacy and safety of ultrasonic bone curette‐assisted dome‐like laminoplasty in the treatment of ossification of longitudinal ligament (OPLL) involving C_2_.

**Methods:**

A total of 64 patients with OPLL involving C_2_ level were enrolled. Thirty‐eight patients who underwent ultrasonic bone curette‐assisted dome‐like laminoplasty were defined as ultrasonic bone curette group (UBC), and 28 patients who underwent traditional high‐speed drill‐assisted dome‐like laminoplasty were defined as high‐speed drill group (HSD). Patient characteristics such as age, sex, body mass index (BMI), symptomatic duration, and other information like the type of OPLL, the time of surgery, blood loss, C_2_–C_7_ Cobb angle change and complications were all recorded and compared. The Japanese Orthopaedic Association (JOA) score, the nerve root functional improvement rate (IR), and the visual analogue scale (VAS) were used to assess neurological recovery and pain relief. The change of the distance between the apex of ossification and a continuous line connecting the anterior edges of the lamina was measured to assess the spinal expansion extent. The measured data were statistically processed and analyzed using SPSS 21.0 software, and the measurement data were expressed as mean ± SD.

**Results:**

In ultrasonic bone curette (UBC) group and high‐speed drill group (HSD) group, the average time for laminoplasty was 52.3 ± 18.2 min and 76.0 ± 21.8 min and the mean bleeding loss volume was 155.5 ± 41.3 mL and 177.4 ± 54.7 mL, respectively, with a statistically significant difference between the groups. Both groups demonstrated a significant improvement in neurological function. However, the VAS score in UBC group was lower than in HSD group at the 6‐month follow‐up (*P* < 0.05), but there was no significant difference at 1‐year follow‐up. We found that the loss of lordosis was 1.5° ± 1.0° in UBC group, which is significantly lower than that of HSD group at 1‐year follow‐up (3.8° ± 1.2°, *P* < 0.05). According to the change of canal dimension, we found that the expansion extent of the spinal canal in UBC group was similar to that of HSD group (*P* > 0.05). Only one patient in the UBC group and five patients in the HSD group displayed cerebrospinal fluid (CSF) leakag.

**Conclusions:**

With the use of ultrasonic bone curette in OPLL dome‐like decompression, the decompression surgery could be completed relatively safely and quickly. It effectively reduced the amount of intraoperative blood loss and complications, and had better initial recovery of neck pain.

## Graphical abstract

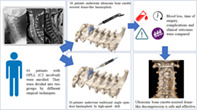

We assessed the efficacy and safety of ultrasonic bone curette‐assisted dome‐like laminoplasty in the treatment of C_2_ involved OPLL. We found that the decompression surgery could be completed relatively safely and with less intraoperative blood loss and fewer postoperative complications by using the ultrasonic bone curette.

## Introduction

Ossification of the posterior longitudinal ligament (OPLL) is a disease that occurs due to ectopic ossification, which is a common cause of cervical compressive myelopathy and neurological injury. It was originally described from occurances in the Japanese and East Asian populations. The prevalence of OPLL has been 1.9%–4.3% among individuals older than 30  years with cervical spine disorders^1^, ^2^. Recent research has demonstrated that the incidence of OPLL has reached 0.1%–1.7% among North Americans and Europeans in recent years^3^. Normally, OPLL took place in the lower cervical spine, but in severe cases like the continuous type and mixed type of OPLL, the upper cervical spine is often involved. Since the narrowest space of the spinal cord is mostly located from C_2_ to C_4_ ^4^, there is a high risk that OPLL patients with their upper cervical spine involved will result in unsatisfied surgical outcomes after the operation due to inadequate decompression with traditional single open‐door laminoplasty, which is most effective in dealing with C_3–7_ cervical stenosis. Recent techniques of dome‐shape decompression at C_2_ level were reported to result in better clinical outcomes in laminoplasty‐treated C_2_‐level‐involved severe OPLL patients. However, whether these techniques were all safe and effective to treat severe OPLL patients remains unknown.

Although the direct removal of the ossified mass can be achieved by anterior approach, the complex anatomic structures around the upper cervical spine caused it to be extremely challenging to attempt direct decompression in OPLL^5^, ^6^. Besides, it has been reported that posterior decompression can significantly lower the occurrence of complications like dysphagia and recurrent laryngeal nerve injury compared to anterior approach^7^. Among all posterior approaches, single open‐door laminoplasty is most widely used by achieving indirect decompression through shifting of the spinal cord posteriorly. Studies showed that the degree of the cervical spinal cord shifting after the decompression post laminoplasty directly affected the postoperative outcome^8^, ^9^. However, the decompression of C_2_ level is often inadequate due to the concern that C_2_ open‐door laminoplasty would induce postoperative kyphosis and axial neck pain as a result of injury to the paraspinal muscle at C_2_ level^10^. Thus, preservation of muscle attachments is an important technique for the laminoplasty to reduce axial neck pain and restriction of cervical range of motion (ROM). In 1989, Matsuzaki *et al*. first reported a dome‐like laminoplasty technique to fully preserve the posterior structure of C_2_ instead of axis laminoplasty^11^. Since then, many studies have reached the consensus that protection of muscle attachments positively contributed to the reduction of postoperative complications^12^, ^13^, ^14^, ^15^, ^16^. However, the traditional procedure of high‐speed drill‐based dome‐like laminectomy in C_2_ has a high risk of spinal cord and nerve injury, which requires careful handling and high technical demand.

More recently, ultrasonic bone curette has been adapted to perform not only endoscopic bone removal over the skull base, but also spine surgeries. Although it has been reported that decompression surgery could be completed more safely and quickly by using ultrasonic bone curette in thoracic surgery when compared with high‐speed drills^17^. However, the efficacy and safety of ultrasonic bone curette in dome‐like laminoplasty have not been reported. Due to the safety concerns of high‐speed drill‐based dome‐like C_2_ laminectomy in C_2_‐involved cervical single open‐door laminoplasty, we hypothesized that using ultrasonic bone curette instead of high‐speed drill in dome‐like C_2_ laminectomy accompanied with single open‐door laminoplasty would be safer and more effective. In order to prove this hypothesis, we: (i) specified the technical procedure of ultrasonic bone curette‐assisted dome‐like single open‐door laminoplasty in the treatment of C_2_‐cervical‐spine‐involved OPLL disease; (ii) compared the clinical outcomes and radiographical outcomes of high‐speed drill‐based or ultrasonic bone curette‐assisted dome‐like single open‐door laminoplasty in treating severe OPLL patients; and lastly (iii) compared the incidence of perioperative complications of the patients in both groups. Through this study, we try to specify the efficacy and safety of ultrasonic bone curette‐assisted dome‐like single open‐door laminoplasty in the treatment of upper‐cervical‐spine‐involved OPLL disease.

## Materials and Methods

### *Ethics Approval and Consent to Participate*

This retrospective study was approved by the ethics committee of our hospital of our university. Written informed consent was obtained by all participants.

### *General Information*

This study retrospectively reviewed 64 patients with C_2_‐involved OPLL from January 2015 to January 2018. Thirty‐eight patients (UBC group: 24 male, 14 female, 64.2 ± 6.6 years old) underwent ultrasonic curette‐assisted C_2_ dome‐like laminoplasty, and twenty‐six patients (HSD group, 18 male, 8 female, 63.4 ± 5.4 years old) underwent traditional high‐speed drill‐assisted C_2_ dome‐like laminoplasty. Inclusion criteria were as follows: (i) patients have typical cervical myelopathy symptoms, and presence of OPLL in C_2_ and downward cervical level was confirmed by imaging, with no significant disc herniation; (ii) diagnosed patients underwent cervical laminoplasty using either ultrasonic bone curette or high‐speed drill‐assisted dome‐like C_2_ laminectomy accompanied with single open‐door laminoplasty; (iii) surgical treated patients with more than 12 months follow‐up. Exclusion criteria were: (i) patients with cervical kyphosis >13° before surgery or K‐line negative; (ii) patients diagnosed with OPLL combined with trauma, tumor, infection, or other systematic diseases; (iii) patients had a mental disorder and other diseases that had an impact on VAS scores. Patient characteristics such as age, sex, BMI, and symptomatic duration were obtained before surgery.

### *Surgical Methods*

**Fig 1:**
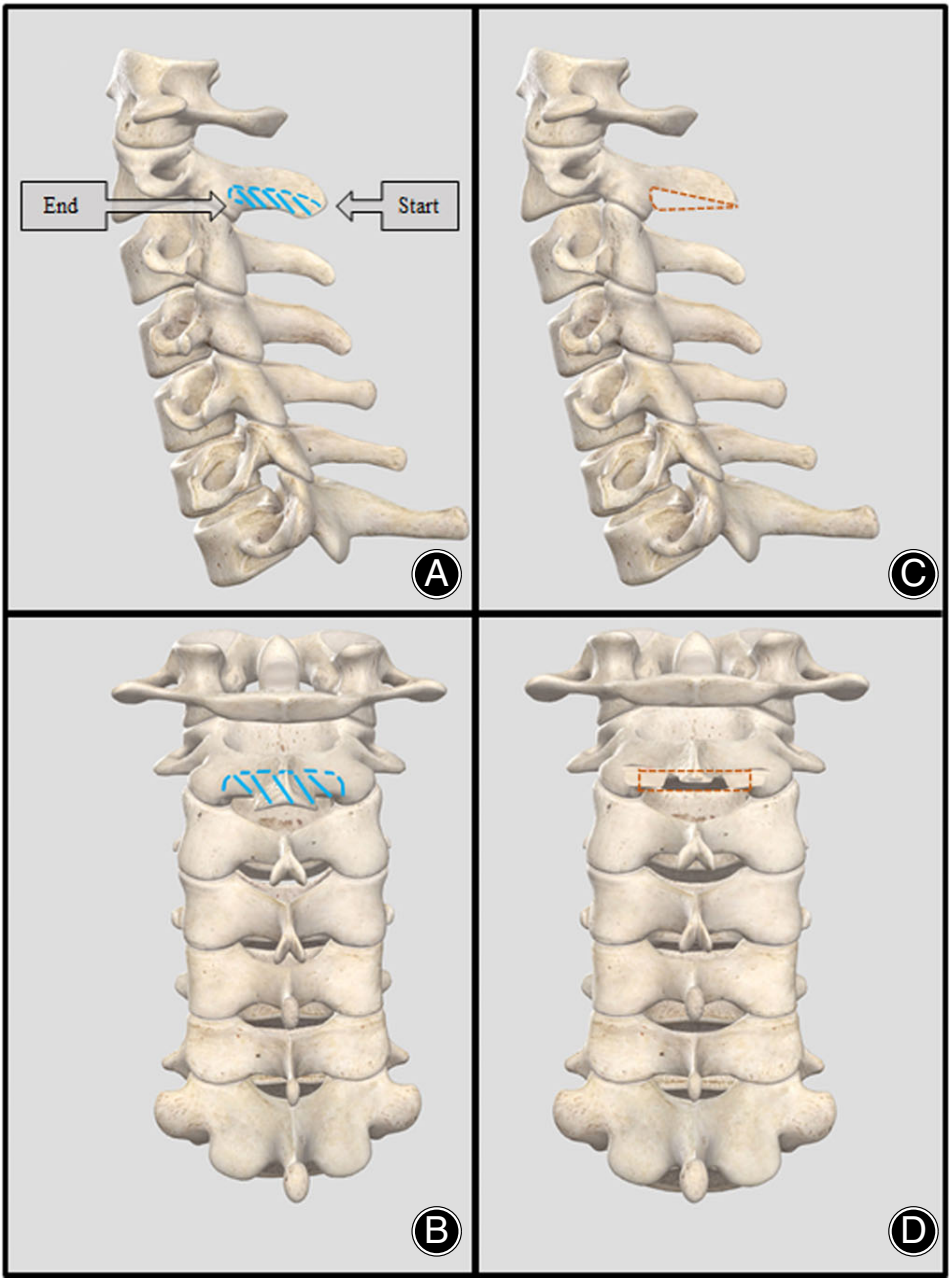
Illustration showing the dome resection area in C _**2**_ using ultrasonic osteotome. (A) The ultrasonic osteotome is applied to achieve the dome‐like resection of C_2_ lamina and spinous process (blue dashed line indicates the area to be cut), note that the posterior part of spinous process is preserved mostly to prevent the removal of the attached muscles. (B) The dome‐shape osteotomy starts from the posterior part of the C_2_ spinous process to the ventral and lower part of C_2_ lamina to expose the posterior part of spinal cord (blue dashed line indicate the area to be cut). (C, D) The illustrations showing the result of C_2_ dome‐like laminectomy, the brown dashed line indicates the area been cut.

**Fig 2:**
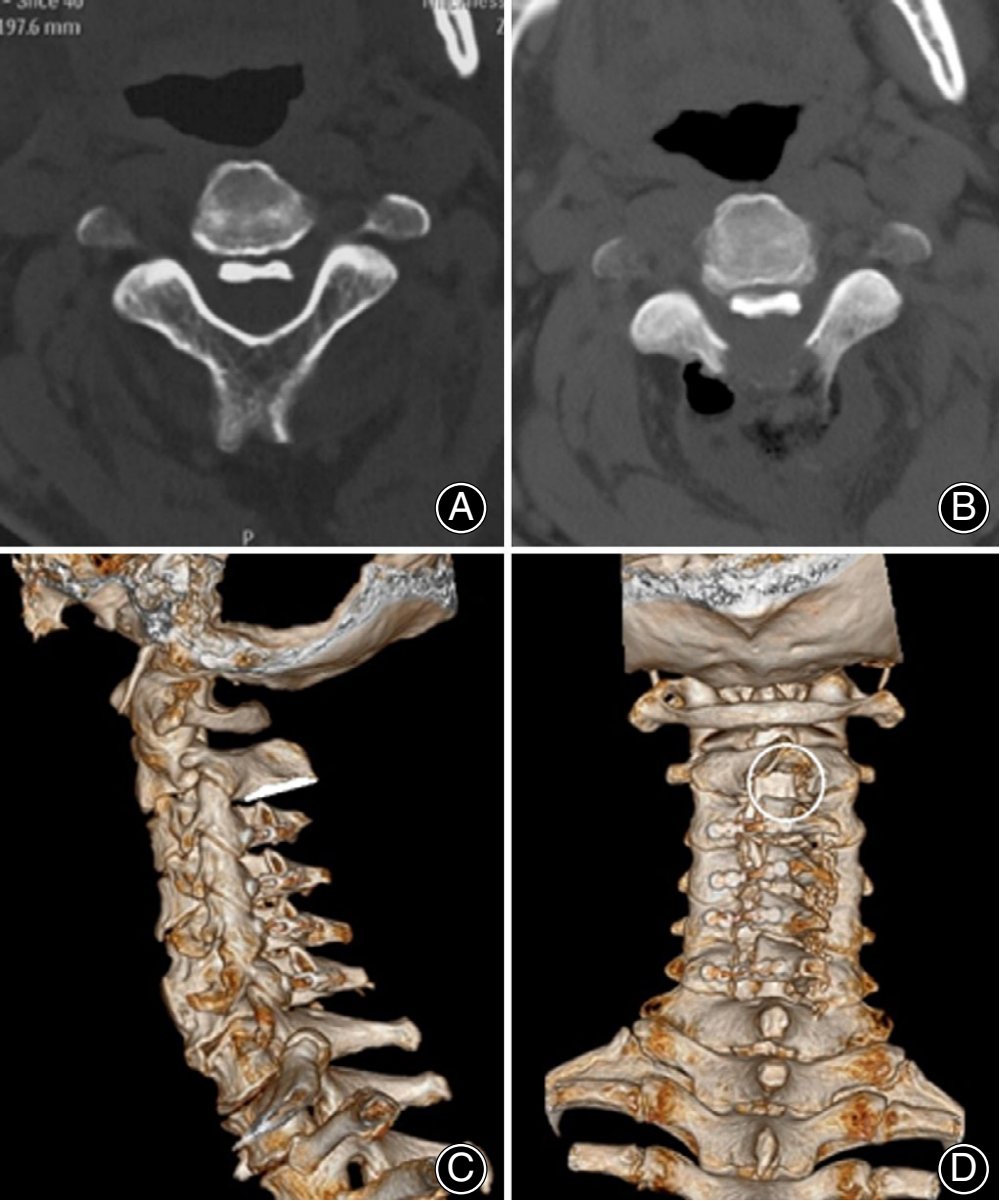
CT scans showing the area of dissected C_**2**_ lamina. The upper two figures showing the same section before (A) and after (B) of the ultrasonic curette‐assisted laminoplasty in C_2_ level. The lower figures showing the reconstructed 3D view of the patient after the curette‐assisted laminoplasty (C, D). Note that resection of flavum ligament is necessary to certify that there has been sufficient space for the spinal cord to shift after the procedure (area is indicated with a white circle in CT reconstructed image in D).

The surgical procedure of dome‐like laminoplasty involves two steps. First, general single open‐door laminoplasty from C_3_ downwards and second, C_2_ dome‐like laminectomy. In the ultrasonic bone curette group (UBC group), the ultrasonic curette is applied to achieve laminectomy of lower 1/3 of the posterior column of C_2_ (axis) from caudal spinous process to the anterior and cranial C_2_ lamina without dissecting the attached muscles (Fig. 1). The resection is performed bilaterally to ensure adequate decompression at C_2_ level. Then Kerrison rongeur is used to remove the flavum ligament to ensure sufficient space for spinal cord to shift back after the procedure (Fig. 2).

In high‐speed drill group (HSD group), the procedure is similar to that of UBC group. Firstly, the single open‐door laminoplasty was performed from C_3_ to lower vertebrae. The ligaments between C_2_ to C_3_ were severed by a rongeur, and, using high‐speed drill bar, a dome‐like groove is made on the caudal surface of the C_2_, leaving up to 5 mm in the posterior wall of the spinal cord–lamina surface. Next, grooves were drilled posterior–anteriorly in a semicircular fashion until the surface of ligamentum flavum was exposed. Finally, a Kerrision rongeur was used to dissect the ligamentum flavum.

### *Radiographic Analysis*

#### *General Measurement Methods*

**Fig 3:**
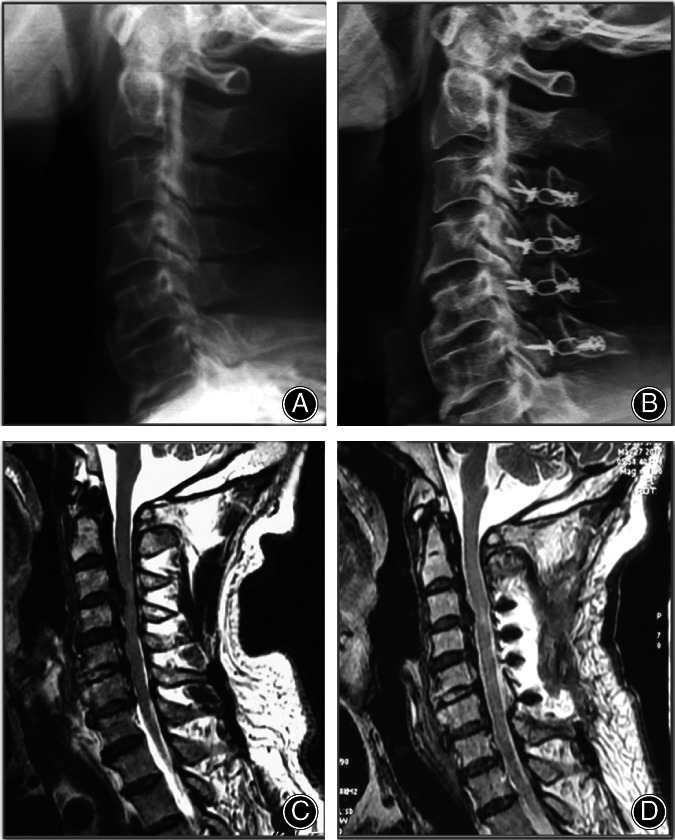
MRI and X‐ray scans showing the shifting of spinal cord after the ultrasonic curette‐assisted laminoplasty. Figure A and C showing the radiograph and MRI sagittal view of the cervical spine before the surgery. B and D showing the radiograph and MRI sagittal view of the cervical spine after the surgery. Note that the spinal cord was loosened in the lower part of C_2_ level.

Cervical spine anteroposterior, lateral, flexion, and extension X‐ray radiographs and cervical spine high‐resolution computed tomography (CT) scans were taken preoperatively and postoperatively. The type of OPLL was confirmed by preoperative CT scans and three‐dimensional reconstruction images (Fig. 3). Measurements were performed by Centricity PACS 4.0 system (GE Healthcare, USA), and the contrast adjustment was made to visualize all cervical spine vertebrae. Two independent clinical research assistants, who were not involved with the study and blinded to all clinical information, performed radiological measurements, and the average values of both observers were used in the present study.

#### *Cervical Lordosis*

Cervical lordosis was assessed by the C_2_–C_7_ Cobb angle. The Cobb angle from C_2_ to C_7_ was used as a measurement of the cervical alignment, which was defined as the angle formed by the inferior end plates of C_2_ and C_7_ in lateral positioned radiographs.

#### *Postoperative Cobb Angle Change*

The change of the C_2_–C_7_ Cobb angle was calculated. Postoperative alignment change was assessed by comparing the preoperative and postoperative C_2_–C_7_ Cobb angle in standing lateral radiographs and was calculated with the following formula: alignment change (°) = (preoperative C_2_–C_7_ Cobb angle) – (postoperative C_2_–C_7_ Cobb angle).

#### *Spinal Cord Expansion Distance*

Spinal cord expansion distance was calculated by first drawing a continuous line connecting the anterior edges of lamina on axial CT scans and measured the change of the distance between this curve line and the apex of ossification to assess the expansion extent of the spinal cord.

### *Outcome Measurements*

#### *Surgical Information*

The time of operation and intraoperative bleeding volume were calculated by two residents. In addition, intraoperative complication such as dural tear was recorded.

#### *Neurological Function Assessment*

The Japanese Orthopaedic Association (JOA) scoring system was adapted to evaluate the neurological function. We use nerve function improvement rate (IR) to assess the symptom improvement, which was calculated as IR = (6 months after surgery JOA scores‐preoperative JOA scores) / (17‐preoperative JOA scores) × 100%. Two independent clinical research assistants, who were not involved in the study and blinded to all clinical information, performed the assessments, and the average values of both observers were used in the present study.

#### *Neck Pain Assessment*

The neck pain was measured by the visual analogue scale (VAS) system. Two independent clinical research assistants, who were not involved in the study and blinded to all clinical information, performed the assessments, and the average values of both observers were used in the present study.

### *Complications*

Postoperative cerebrospinal fluid (CSF) leakage, the event of axial neck pain, and C5 nerve root palsy were recorded in both groups.

### *Statistical Analysis*

Data analyses were analyzed using SPSS version 20 for Windows (SPSS, Inc., Chicago, IL, USA). Data are presented as the number of subjects in each group or mean ± SD. Each independent variable, such as age, sex, symptom duration, follow‐up period, JOA score, VAS score, C_2_–C_7_ cobb angle, and C_2_–C_7_ ROM was compared between the two groups using the Mann–Whitney U test for continuous variables, and the *χ2* test or Fisher exact test for categorical variables. A statistically significant difference was set at *P* value <0.05.

## Results

### *General Results*

Thirty‐eight patients who underwent ultrasonic curette‐assisted C_2_ dome‐like laminoplasty were defined as group UBC, and twenty‐six patients who underwent a traditional single open‐door laminoplasty using high‐speed drills were defined as group HSD. There was no significant difference among age, sex, BMI, symptomatic duration, and the type of OPLL between the two groups (Table 1, *P* > 0.05). All patients enrolled had a follow‐up period of longer than 1 year.

**TABLE 1:** Comparison of patient demographics and characteristics

	UBC Group	HSD Group	*P* value
Age	64.2 ± 6.6	63.4 ± 5.4	0.68
Gender (Male: Female)	(24:14)	(18:8)	0.92
BMI	26.1 ± 3.0	25.5 ± 2.5	0.40
Type of OPLL			0.56
Local	0	0	‐ ‐ ‐ ‐
Segmental	1	2	
Continuous	8	4	
Mixed	29	22	
Symptom duration (Months)	10.8 ± 10.4	12.6 ± 12.2	0.63
Follow‐up period (Months)	18.2 ± 6.8	17.8 ± 6.2	0.38

*BMI*, body mass index; *HSD Group*, traditional single open‐door laminoplasty using high‐speed drills treated patients; *OPLL*, ossification of posterior longitudinal ligament; *UBC Group*, ultrasonic curette‐assisted C_2_ dome‐like laminoplasty treated patients. Values are expressed as the mean ± standard deviation. “‐” represents values were not compared for differences.

### *Surgical Outcomes*

The average time of operation was 52.3 ± 18.2 min in UBC group, which is significantly lower (*P* = 0.04) than that of HSD group (76.0 ± 21.8 min, Table 2). In addition, the mean bleeding loss volume was 155.5 ± 41.3 mL and 177.4 ± 54.7 mL, respectively, with no statistical significance between the groups (*P* = 0.21). Only one patient in the UBC group compared to four patients in the HSD group (*P* = 0.04) had CSF leakage. No other complications were found in both groups during the surgery.

**TABLE 2:** Comparison of surgical information between ultrasonic bone curette and high‐speed drill‐assisted laminoplasty

	UBC Group	HSD Group	*P* value
Time of surgery (min)	52.3 ± 18.2	76.0 ± 21.8	0.04*
Bleeding loss volume (mL)	155.5 ± 41.3	177.4 ± 54.7	0.21
Spinal cord expansion (mm)	3.9 ± 1.1	4.2 ± 1.2	0.32
CSF leakage	1	4	0.04*

Values are expressed as the mean ± standard deviation. *HSD Group*, traditional single open‐door laminoplasty using high‐speed drills treated patients; *UBC Group*, ultrasonic curette‐assisted C_2_ dome‐like laminoplasty treated patients. Spinal cord expansion was compared using postoperative CT to the data before the surgery.

* *P* < 0.05.

### *Radiographic Results*

#### *General Results*

All patients showed good neural decompression from postoperative radiographic analysis. The dome‐shape laminectomy in C_2_ level resulted in an average of 3.62 ± 1.3 mm width in UBC group and 3.3 ± 1.2 mm in HSD group with no significant differences (*P* = 0.27). The postoperative change of spine canal mean width was 3.9 ± 1.1 mm in UBC group and 4.2 ± 1.2 mm in HSD group, which also showed no significant differences (*P* = 0.32, Table 2). The occupation rate (OR) of osteophytes in C_2_ level has changed from 42.3 ± 5.6 (%) before surgery to 17.5 ± 3.8 (%) after surgery in UBC group, and 45.6 ± 4.3 (%) to 18.2 ± 3.6 (%) in HSD group, no significant differences were found between the groups (*P* > 0.05).

#### *C_2_–C_7_ Cobb Angle*

The preoperative C_2_–C_7_ cobb angle was 12.2° ± 2.7° in UBC group and 11.9° ± 2.5° in HSD group, respectively, with no statistical difference between the groups (*P* = 0.52). At the final follow‐up, the C_2_–C_7_ cobb angle was 10.8° ± 2.3° in UBC group and 8.2° ± 2.2° in HSD group. Although the mean cobb angle was lower in HSD group, these is no significant differences between the groups (*P* = 0.09).

#### *Postoperative Cobb Angle Change*

The loss of lordosis in the two groups also showed no significant differences (*P* = 0.06), with 1.5° ± 1.0° in UBC group and 2.8° ± 1.2° in HSD group, respectively (Table 3).

**TABLE 3:** Postoperative clinical data comparison of the patients

	UBC Group	HSD Group	*P* value
JOA score			
Before surgery	10.3 ± 2.5	9.8 ± 2.7	0.52
6 months after surgery	14.2 ± 2.2	13.9 ± 2.4	0.49
IR (%)	58.4 ± 9.2	56.5 ± 8.9	0.45
VAS			
Before surgery	5.2 ± 1.5	5.4 ± 1.3	0.43
6 months after surgery	2.8 ± 1.6	3.9 ± 1.7	0.03*
The final follow‐up	2.1 ± 1.5	2.9 ± 1.6	0.21
C_2‐7_ cobb angle (degree)			
Before surgery	12.2 ± 2.7	11.9 ± 2.5	0.52
6 months after surgery	11.5 ± 2.8	10.1 ± 2.7	0.19
The final follow‐up	10.8 ± 2.3	8.2 ± 2.2	0.09
The loss of lodorsis	1.5 ± 1.0	2.8 ± 1.2	0.06
Postoperative Complications (number of patients (percentage))			
Axial neck pain	2 (5.3%)	6 (23.0%)	0.05*
C_5_ nerve root palsy	1 (2.6%)	4 (15.4%)	0.15
CSF leakage	1 (2.6%)	5 (19.2%)	0.04*

Values are expressed as the mean ± standard deviation. *CSF*, cerebrospinal fluid; *HSD*, high‐speed drill; *IR*, improvement rate; *JOA*, Japanese Orthopaedic Association; *VAS*, Visual Analog Scale; *UBC*, ultrasonic bone curette.

* *P* < 0.05.

### *Clinical Outcomes*

#### *Neurological Function Assessment*

Both groups demonstrated a significant improvement in neurological function, and there were no significant differences between the JOA score (14.2 ± 2.2 in UBC group and 13.9 ± 2.4 in HSD group respectively, Table 3, *P* = 0.49) and the IR (58.4% ± 9.2% in UBC group and 56.5% ± 8.9% in HSD group respectively, Table 3, *P* = 0.45) between the two groups.

#### *Neck Pain Assessment*

In UBC group, the average VAS score decreased from 5.2 ± 1.5 to 2.8 ± 1.6 at the 6‐month follow‐up, and the average VAS score in HSD group declined from 5.4 ± 1.3 to 3.9 ± 1.7. The VAS score of a 6‐month follow‐up had a significant difference between the two groups (Table 3, *P* = 0.03). However, the VAS score of the final follow‐up did not show a significant difference (*P* = 0.21).

### *Complications*

After the surgery, two patients in UBC group and six patients in HSD group experienced sustained axial pain (*P* = 0.05), and all recovered within 1‐year post‐operation. One patient in UBC group and five patients in HSD group experienced cerebrospinal fluid (CSF) leakage during and after the surgery (*P* = 0.04). One patient in UBC group and four patients in HSD group experienced C_5_ palsy after the surgery (*P* = 0.15), but recovered within 4 months, and no significant differences were found between the two groups (Table 3). There was no nerve root injury, postoperative hematoma, or other complications that occurred after operation.

## Discussion

### *Essentiality of Preserving C_2_ Muscle Attachments*

Muscles and ligaments attached to the lamina and spinous process are recognized as the components of neck dynamic equilibrium. However, it is unavoidable to dissect paraspinal muscles in laminoplasty. A wide range of decompression results in excessive posterior structure destruction. For most patients, a C_3_–C_7_ laminoplasty can achieve satisfied indirect decompression due to a spinal cord shift. Nevertheless, in some cases, considering the C_2_ segment was severely compressed, or with a high‐intensity signal on T2‐weighted magnetic resonance imaging, C_2_ decompression was recommended, which can cause the sacrifice of cervical deep extensor. Some studies suggested that, due to the expansion of the decompression range, the backward shift distance of the spinal cord for C_2_–C_7_ surgery was indeed increased compared with that of C_3_–C_7_ surgery^18^, ^19^. But posterior cervical deep extensors, especially the semispinalis cervicis, play an important role in maintaining the lordosis and alignment of the cervical vertebrae. Besides, many researchers have attributed axial neck pain and loss of ROM to the destruction of C_2_ muscle attachments^20^, ^21^. On the other hand, A meta‐analysis suggested that laminoplasty preserving the C_2_ posterior deep extensor muscle could decrease the atrophy rate and reduce the incidence of postoperative axial symptoms^22^. Thus, to address this problem, several modified surgical techniques of laminoplasty have been reported. The C_2_ canal space is wider at the cranial and narrower at the caudal end; thus, ventral decompression of C_2_ lamina may provide extra space for spinal cord shift. This theory was first proved by Matsuzaki *et al*. in 1989, as their dome‐like decompression of C_2_ lamina was able to fully preserve the posterior deep extensor muscle. However, space for surgeons to perform this surgical technique using a high‐speed drill is quite limited, which can result in a high risk of dural tear.

### *Safety of Ultrasonic Bone Curette in Dome‐Like Laminoplasty*

Although the ultrasonic aspirator was introduced for removal of dental plaque in 1947, the application of ultrasonic osteotome has turned out to be a versatile, safe, and efficient method for bone removal within spine surgery in recent years, especially in the thoracic spine and lumbar spine^23^, ^24^. The previous study has suggested that the ultrasonic bone curette had some technical advantages in cutting the laminae. For instance, the device is lightweight, requires the use of only one hand, features both irrigation and aspiration attachments, and allows for the placement of cotton buffers^25^. Because there was no rotational movement, the tip of the device was much more stable. Besides, ultrasonic bone curette produces less heat compared with a high‐speed drill. While the curette is being used, the handpiece end is cooled by the automatic irrigation of saline water. There is less danger of causing thermal injury to the surrounding important neural and vascular structures. It also can reduce the bleeding and shorten the operation time during the process of cutting the lamina^26^. In this present study, we further proved that using ultrasonic bone curette could reduce blood loss and the time of operation in dome‐like laminoplasty. There was only one case of CSF leakage in the ultrasonic bone curette‐assisted laminoplasty group, which showed merit over the traditional high‐speed drill in dealing with the dome cutting (five patients in this group were found to have CSF). Also, the incidence of other complications was much lower than that in the high‐speed drill group. However, care must be taken to avoid iatrogenic dural tears. We suggested using covering cotton patties on the surface of dura to prevent intraoperative dural tear.

### *Clinical Outcomes of Ultrasonic Bone Curette‐assisted Dome‐like Laminoplasty*

In this study, both groups showed significant improvement in neurological function. However, the VAS score in ultrasonic bone curette group was significantly lower than that in the high‐speed drill group at 6‐month follow‐up. As the previous study demonstrated, the injury of the C_2_ spinous process could be a factor of axial neck pain. We considered that the reason of why VAS score was significantly higher in high‐speed drill group might be due to the substantial exposure of C_2_ laminae using a high‐speed drill. Nevertheless, no significant difference was found at the final follow‐up. Although kyphosis did not occur in both groups, we observed some loss of lordosis at the final follow‐up in high‐speed drill group, which may be caused by the over‐exposure of the C_2_ laminae, which sacrificed more muscle attachments than ultrasonic bone curette group in order to maintain safe surgery.

It is the first time that we have presented a change of the distance between a continuous line connecting the anterior edges of the lamina and the apex of ossification to assess the expansion extent of the spinal cord on axial CT scans. As a result, the mean change of distance was 3.9 ± 1.1 mm postoperatively in UBC group, which is similar to high‐speed drill group. The change of distance demonstrated that the distance of the spinal cord shift is approximately 4 mm after dome‐like laminoplasty, which resembles to that of traditional single open‐door laminoplasty from C_2_–C_7_ ^27^.

### *Limitation*

This is a retrospective study, meaning that inherent biases may exist that interfere with the results. The sample size in this study was relatively small and needs further comparison with large‐scale cases. Hence, large sample size and long‐term follow‐up research is needed for further confirmation.

### *Conclusion*

Our findings have shown that the ultrasonic curette‐assisted dome‐like decompression surgery could be completed relatively safely and quickly. It effectively reduced the amount of intraoperative blood loss and complications and had better initial recovery of neck pain.

## Authors' Contributions

Study conception and design: BS, CX, YL; Acquisition, analysis and/or interpretation of data: BS, CX, YL, SW, YZ; Drafting/revision of the work for intellectual content and context: HW, MQ, XS, WY; Final approval and overall responsibility for the published work: YL, WY. All authors read and approved the final manuscript.
